# Mean platelet volume may predict early clinical outcome after coronary artery bypass grafting

**DOI:** 10.1186/1749-8090-8-91

**Published:** 2013-04-16

**Authors:** Ertekin Utku Unal, Anil Ozen, Sabit Kocabeyoglu, Ahmet Baris Durukan, Sercan Tak, Murat Songur, Umit Kervan, Cemal Levent Birincioglu

**Affiliations:** 1 Department of Cardiovascular Surgery, Turkey Yuksek Ihtisas Hospital, Ankara, Turkey; 2 Department of Cardiovascular Surgery, Medicana International Hospital, Ankara, Turkey

**Keywords:** Coronary artery bypass, Platelet function tests, Hematocrit, Postoperative complications, Treatment outcome

## Abstract

**Background:**

An elevated mean platelet volume is associated with increased platelet activation and thus may predict thrombotic events. The goal of this study was to investigate the association of the mean platelet volume and the major adverse events after coronary artery bypass surgery.

**Methods:**

Baseline clinical details and preoperative hematologic parameters were obtained prospectively in 205 consecutive patients undergoing coronary artery bypass surgery. Postoperative mortality and major adverse events were recorded in the early postoperative period (median of 72 days, interquartile range 58.5-109 days).

**Results:**

Combined adverse events occurred in 37 patients (18.0%) during the early follow-up. The preoperative mean platelet volume and hematocrit levels were found to be associated with postoperative adverse events (p<0.001 for both variables). In multivariate logistic regression models, the preoperative mean platelet volume and hematocrit levels were strong independent predictors of combined adverse events after surgery (respectively OR 1.89, p=0.037; OR 0.87, p=0.011). After receiver-operating-characteristics curve analysis, using a cut-point of 8.75 fL, the preoperative mean platelet volume level predicted adverse events with a sensitivity of 54% and specificity of 70%. In a further model with cut-off points, higher preoperative mean platelet volume levels remained a powerful independent predictor of postoperative myocardial infarction (OR 3.60, p=0.013) and major adverse cardiac events (OR 2.53, p=0.045).

**Conclusions:**

An elevated preoperative mean platelet volume is associated with an adverse outcome after coronary artery bypass grafting. In conclusion, we can say that mean platelet volume is an important, simple, readily available, and cost effective tool and can be useful in predicting the postoperative adverse events in patients undergoing coronary artery bypass grafting.

## Background

Coronary artery bypass grafting (CABG) is the definitive surgical treatment of the coronary artery disease and can be performed with a low incidence of morbidity and mortality. European System for Cardiac Operative Risk Evaluation (EuroSCORE) is widely accepted and used for routine application of risk stratification in adult cardiac surgery [1]. However, there is always a tendency in search of more and more reliable and additional predictors.

Mean platelet volume (MPV) is a marker of platelet size and activation. Increased MPV reflects active and large platelets. MPV level is probably the most extensively used platelet activation marker. Activated platelets play a role in the pathogenesis of coronary heart disease [2]. Increased MPV level, an indicator of larger and more reactive platelets, has been reported to be associated with some cardiovascular diseases [3-6]. Mean platelet volume has also been shown to be associated with late saphenous vein graft disease after CABG [7]. Little is known, however, about the association of the MPV levels with the outcome of CABG [8].

Decreased hematocrit or hemoglobin level is a clinically important condition and it is not an uncommon finding in patients undergoing CABG. Many studies have established the association of anemia with increased perioperative morbidity and mortality [9-11]. Patients undergoing CABG are the most sensitive to low levels of hematocrit because of their limited capacity of coronary level [9].

We hypothesized that an elevated MPV level would be associated with an increased incidence of adverse events after CABG surgery. In addition, we aimed to determine the predictive value of the preoperative MPV and hematocrit levels for the incidence of post-CABG adverse events.

## Methods

From December 2011 to March 2012, 240 consecutive patients underwent isolated coronary artery bypass grafting at our institution. Five patients who underwent redo surgery, 27 patients who underwent off-pump surgery and 3 patients with hematologic problems were excluded. The study population was composed of 205 patients. This study complies with the Declaration of Helsinki and ethical approval was granted by the Research Committee of Turkey Yuksek Ihtisas Hospital (Ankara, Turkey). Informed consent was obtained from all patients.

Exclusion criteria included, (1) emergent surgery, (2) redo CABG, (3) off-pump CABG, (4) myocardial infarction within a week, (5) preoperative severe anemia (hemoglobin level < 10 g/dl and/or hematocrit level < 30%), (6) acute or chronic infections, (7) known malignancies, and (8) other hematological problems.

The demographic and baseline clinical data, including New York Heart Association functional class, cardiovascular risk factors, medical history, the EuroSCORE II were obtained prospectively. Preoperative antecubital venous blood samples in EDTA-containing tubes were used for the baseline data. Complete blood count analysis, including differential leukocyte count, was measured using an automated flow counter (Sysmex SE 9500, Roche Diagnostics, Mannheim, Germany). Hemoglobin and hematocrit levels mean corpuscular volume, mean corpuscular hemoglobin, red cell distribution width, platelet count, MPV levels, total white cell, neutrophil and lymphocyte counts were recorded and neutrophil to lymphocyte (N/L) ratio was calculated. The expected values for MPV in our hematology laboratory ranged from 6.1 to 8.9 fl.

Combined adverse events were defined as all-cause mortality, postoperative myocardial infarction (MI), reoperation due to hemodynamic instability, early repeat revascularization (percutaneous intervention or re-CABG), prolonged ventilation time (>24 hours), and rehospitalization because of any cardiac indication at the follow-up period.

Major adverse cardiac events (MACE) were defined as postoperative MI, reoperation due to hemodynamic instability, early repeat revascularization and in-hospital mortality. Postoperative MI was defined as creatine kinase myocardial band above 5 times the upper limit of normal or high troponin I levels (troponin I level above 15 ng/ml at postoperative day 1 and above 35 ng/ml at postoperative day 2) and/or new electrocardiographic changes [12]. In-hospital mortality was the all-cause mortality during the hospitalization period.

### Statistical analyses

Continuous variables were tested for normal distribution by Kolmogorov-Smirnov test. Normally distributed continuous variables were expressed as ‘mean values ± standard deviation (SD)’ or median values with the interquartile range if not normally distributed. Categorical variables were expressed as numbers and percentages. Demographic characteristics, perioperative variables and calculated values were compared using “independent samples *t*-test” or “Mann–Whitney-*U* test” for continuous variables and “chi-square test” or “Fisher’s exact test” for categorical variables. To compare groups based on quartiles of MPV and hematocrit levels, one-way ANOVA test was used. Correlations were assessed using Pearson’s correlation test. Receiver operating characteristic curve analysis was used to determine the optimum cut-off levels of the preoperative MPV and hematocrit level to predict post-CABG adverse events. The odds ratios and 95% confidence intervals were estimated with different logistic regression models that were created to determine independent predictors of post-CABG adverse events. A p value < 0.05 was considered statistically significant. All statistical analyses were performed using the SPSS statistical software (SPSS for Windows 15.0, Inc., Chicago, IL, USA).

## Results

### Study population

Baseline characteristics are listed in Table 1. The study population was predominantly male (82%) and composed of 205 patients with a mean age of 60.9 ± 10.3 years. During a median of 72 days (interquartile range 58.5-109 days) follow-up for all patients, 8 patients (3.9%) died during their hospital stay after surgery and 4 of them occurred within the first 30 days of surgery. There was no early post-discharge mortality. During the follow-up combined adverse events occurred in 37 patients (18.0%). There was a tendency of a lower values of EF as the preoperative MPV increased between the groups based on quartiles of the preoperative MPV level (p=0.016). However, older and female patients tended to have lower preoperative hematocrit level (respectively p=0.030 and p<0.001).

**Table 1:** Clinical and hematologic characteristics of patients and univariable association to post-CABG adverse events

**Characteristic**	**All patients (N=205)**	**Adverse event**		
		**No (n=168)**	**Yes (n=37)**	***p value***
Patient characteristics				
Age (years)	60.9 ± 10.3	60.4 ± 10.6	63.4 ± 8.5	0.111^*^
Male	168 (82.0%)	142 (84.5%)	26 (70.3%)	0.041^†^
HT	129 (62.9%)	103 (61.3%)	26 (70.3%)	0.307^†^
HL	112 (54.6%)	94 (56.0%)	18 (48.6%)	0.419^†^
DM	73 (35.6%)	61 (36.3%)	12 (32.4%)	0.656^†^
COPD	35 (17.1%)	28 (16.7%)	7 (18.9%)	0.742^†^
EF (%)	55.0 (50.0-60.0)	55.0 (50.0-60.0)	50.0 (45.0-55.0)	0.010^‡^
NYHA functional class				0.011^†^
1	146 (71.2%)	126 (75.0%)	20 (54.1%)	
2-3	59 (28.8%)	42 (25.0%)	17 (45.9%)	
EuroSCORE	0.93 (0.67-1.51)	0.84 (0.62-1.41)	1.31 (0.85-1.86)	0.001^‡^
Preoperative blood results				
Renal function				
Creatinine (mg/dl)	0.93 (0.80-1.07)	0.94 (0.81-1.08)	0.90 (0.74-0.99)	0.307^‡^
Estimated GFR (ml/min per 1.73 m^2^)	94.4 ± 33.5	95.0 ± 34.5	92.1 ± 28.7	0.634^*^
Hematologic paramaters				
Hemoglobin (g/L)	14.4 ± 1.6	14.6 ± 1.5	13.4 ± 1.5	<0.001^*^
Hematocrit (%)	43.5 ± 5.2	44.1 ± 5.3	40.6 ± 4.2	<0.001^*^
MCV (fL)	89.1 ± 4.9	89.1 ± 5.1	89.3 ± 4.1	0.848^*^
MCH (pg)	29.7 ± 1.9	29.7 ± 1.9	29.6 ± 1.8	0.722^*^
RDW (%)	13.6 (13.1-14.2)	13.5 (13.1-14.2)	13.6 (13.1-14.3)	0.274^‡^
Platelet count (x10^9^/L)	268 ± 67	270 ± 67	262 ± 66	0.502^*^
MPV (fL)	8.50 ± 0.78	8.44 ± 0.76	8.80 ± 0.80	0.009^*^
Total white cell count (x10^9^/L)	8.27 ± 1.93	8.29 ± 2.00	8.17 ± 1.63	0.741^*^
Neutrophil count (x10^9^/L)	5.24 ± 1.64	5.23 ± 1.67	5.25 ± 1.48	0.955^*^
Lymphocyte count (x10^9^/L)	2.16 ± 0.69	2.19 ± 0.68	2.03 ± 0.72	0.211^*^
Neutrophil-to-lymphocyte ratio	2.32 (1.86-3.19)	2.30 (1.81-3.15)	2.61 (1.95-3.52)	0.228^‡^
Operative data				
Bypass time (min)	96.7 ± 36.0	93.0 ± 35.1	113.8 ± 35.5	0.001^*^
Cross-clamp time (min)	64.0 ± 25.5	62.9 ± 26.2	69.2 ± 21.8	0.173^*^
Number of bypass grafts	3.0 (3.0-4.0)	3.0 (2.0-4.0)	4.0 (3.0-4.0)	0.018^‡^
Length of stay in ICU (day)	1 (1–2)	1 (1–2)	3 (1–6)	<0.001^‡^
Length of stay in hospital (day)	6 (5–7)	6 (5–7)	9 (7–18)	<0.001^‡^

*HT*: Hypertension, *HL*: Hyperlipidemia, *DM*: Diabetes mellitus, *COPD*: Chronic obstructive pulmonary disease, *EF*: Ejection fraction, *NYHA*: New York Heart Association, *MCV*: Mean corpuscular volume, *MCH*: Mean corpuscular hemoglobin, *RDW*: Red cell distribution width, *MPV*: Mean platelet volume, *ICU*: Intensive care unit.

Data are expressed as mean ± SD for normally distrubuted data and median (interquartile range) for skewed data or count (percentage) for categorical variables. ^*^ Independent-samples *t* test, ^†^ Chi-square test, ^‡^ Mann–Whitney *U* test.

### Univariate analyses

The patients who developed post-CABG adverse event were predominantly male and significantly had lower EF, greater preoperative risk score and greater NYHA class than those who had an uneventful course. The preoperative hemoglobin and hematocrit levels were lower in the patients who developed post-CABG adverse event. However, the MPV level was greater. No difference in the white cell count, differential leukocyte count and N/L ratio were found between the 2 groups. Cardiopulmonary bypass time and number of bypass grafts were significantly greater in the adverse event group (Table 1).

Receiver operating characteristic curves for MPV and hematocrit levels showed the relation with adverse events after CABG. The area under curve for the preoperative MPV levels was 0.63 (95% CI 0.53 to 0.73; p=0.013). Using a cut-point of 8.75 fL, the preoperative MPV level predicted adverse events with a sensitivity of 54% and specificity of 70%. The OR for patients with a MPV level greater than 8.75 fL was 2.78 (95% CI 1.34 to 5.74. p=0.005. *x*^2^=7.96).

The area under curve for preoperative hematocrit levels was 0.70 (95% CI 0.62 to 0.79; p< 0.001). Using a cut-off value of 41.5%, the preoperative hematocrit level correlated with the incidence of adverse events with a sensitivity of 57% and specificity of 72%. Patients with a hematocrit level lower than this value had a threefold increased risk of developing adverse events (OR 3.38, 95% CI 1.62 to 7.03; p=0.001. *x*^2^=11.33).

The other hematological parameters such as MCV, MCH, RDW, platelet count, total white cell, neutrophil and lymphocyte counts and N/L ratio were found not to be associated with post-CABG adverse events and mortality (Table 1).

### Multivariate analyses

The preoperative MPV levels and platelet counts moderately correlated (r = −0.26, p<0.001). There was a strong correlation between hematocrit and hemoglobin levels as they are closely related measures (r = 0.972, p<0.001). Also there was a strong correlation between CPB time and cross-clamp time (r = 0.869, p<0.001) and also with number of bypass grafts (r= 0.672, p<0.001). The N/L ratio was measured and related to the neutrophil and lymphocyte counts. Therefore, the preoperative MPV level, hematocrit level, N/L ratio and CPB time were entered in the subsequent multivariate regression models. All preoperative variables listed in Table 1 except EuroSCORE, and operative variables were included in the first model. EuroSCORE was excluded because of its dependency on these variables. In this model, preoperative MPV and hematocrit levels and time on bypass were the independent predictors of post-CABG adverse event (Table 2). In an another model with the significant variables (male gender, EF, NYHA class or EuroSCORE and time on bypass) at the univariate analysis, preoperative MPV and hematocrit levels along with time on bypass were found to be predictors of post-CABG adverse event (respectively; OR 1.74 per unit, 95% CI 1.04 to 2.89, p=0.034; OR 0.88 per unit, 95% CI 0.80 to 0.97, p=0.009; OR 1.20 per 10 min, 95% CI 1.07 to 1.35, p=0.002). In a further model with the EuroSCORE, preoperative MPV and hematocrit levels remained the independent predictors of post-CABG adverse event (respectively; OR 1.83 per unit, 95% CI 1.11 to 3.02, p=0.017; OR 0.87 per unit, 95% CI 0.79 to 0.95, p=0.003). In similar models, a preoperative MPV level ≥ 8.75 fL and hematocrit level ≤ 41.5% were associated with three-fold increased risk of post-CABG adverse events separately (respectively; OR 3.31, 95% CI 1.47 to 7.42, p=0.004; OR 0.33 per unit, 95% CI 0.15 to 0.72, p=0.003).

**Table 2:** Multivariable predictors of post-CABG adverse events

**Variable**	**OR**	**95% CI**	***p value***
Preoperative hematocrit level (%)	0.87	0.78-0.97	0.011
Preoperative MPV level (fL)	1.89	1.04-3.44	0.037
CPB time (per 10 min)	1.22	1.08-1.39	0.002

*MPV*: mean platelet volume, *CPB*: cardiopulmonary bypass.

### Secondary analyses

The outcomes of the surgery were detailed into varying groups as explained earlier. When stratified for the pre-determined cut-off values of preoperative hematocrit levels (> 41.5% vs. ≤ 41.5%), combined adverse events (p=0.001), postoperative MI (p=0.048), MACEs (p=0.017), prolonged ventilation (p=0.034), re-hospitalization (p=0.019), prolonged ICU stay (p=0.005) and prolonged hospital stay (p=0.002) were found to be associated with the hematocrit cut-off value. However, only combined adverse events (p=0.005) and postoperative MI (p=0.019) were found to be associated with the MPV cut-off value (< 8.75 fL vs. ≥ 8.75 fL) (Table 3).

**Table 3:** Outcome parameters according to preoperative MPV level and hematocrit levels

**Outcome**	**Preoperative MPV level**			**Preoperative hematocrit level**		
	**< 8.75 fL (n=135)**	**≥ 8.75 fL (n=70)**	***p value***	**> 41.5% (n=137)**	**≤ 41.5% (n=68)**	***p value***
Combined adverse events (n=37)	17 (12.6%)	20 (28.6%)	0.005^*^	16 (11.7%)	21 (30.9%)	0.001^*^
Postoperative MI (n=21)	9 (6.7%)	12 (17.1%)	0.019^*^	10 (7.3%)	11 (16.2%)	0.048^*^
Mortality (n=8)	6 (4.4%)	2 (2.9%)	0.718^†^	4 (2.9%)	4 (5.9%)	0.444^†^
MACE (n=26)	13 (9.6%)	13 (18.6%)	0.068^*^	12 (8.8%)	14 (20.6%)	0.017^*^
Prolonged ventilation (n=13)	9 (6.7%)	4 (5.7%)	1.000^†^	5 (3.6%)	8 (11.8%)	0.034^†^
Re-hospitalization (n=17)	8 (5.9%)	9 (12.9%)	0.088^*^	7 (5.1%)	10 (14.7%)	0.019^*^
Prolonged ICU stay (days) (n=31)	16 (11.9%)	15 (21.4%)	0.070^*^	14 (10.2%)	17 (25.0%)	0.005^*^
Prolonged hospital stay (days) (n=44)	27 (20.0%)	17 (24.3%)	0.478^*^	21 (15.3%)	23 (33.8%)	0.002^*^

*MPV*: Mean platelet volume, *MI*: Myocardial infarction, *MACE*: Major adverse cardiac events, *ICU*: Intensive care unit.

^*^ Chi-square test, ^†^ Fisher’s exact test.

Multivariable models for different outcomes (including the preoperative MPV and hematocrit level as a categorical variable around described cut-off values, EuroSCORE and CPB time) were created. The preoperative MPV level remained a powerful independent predictor of postoperative MI (OR 3.60, 95% CI 1.31 to 9.85, p=0.013) and MACE (OR 2.53, 95% CI 1.02 to 6.31, p=0.045). Conversely, the preoperative hematocrit level remained an independent predictor of prolonged ICU stay (> 2 days) (OR 2.54, 95% CI 1.11 to 5.82, p=0.027) and prolonged hospital stay (> 7 days) (OR 2.48, 95% CI 1.20 to 5.14, p=0.014). The mean length of postoperative hospital stay was 10.3 ± 9.6 days (median 7 days) for patients with hematocrit ≤ 41.5% versus 8.42 ± 13,48 days (median 6 days) for patients with hematocrit > 41.5% (p=0.018).

## Discussion

Risk factors affecting mortality and morbidity following cardiac surgery have been studied for several decades. The risk-stratification models including many variables have been introduced to the clinical use [1,13-15]. The most commonly used risk score system for evaluation of operative mortality is the EuroSCORE for our institution [1]. Although they are widely used, none of them is perfect for the prediction of the outcome. On the other hand, another scoring system, the STS score was published in 2009 [15]. In contrast to the EuroSCORE, the required data entry is more detailed. There is always a great interest to generate a better risk assessment model with additional data that is easily obtained and widely available.

Many studies have assessed the association between the preoperative hemoglobin level and adverse events after CABG [6,16,17]. However there are few studies regarding the relationship between the other hematologic parameters and adverse events after CABG [18,19].

Platelet activity is a major responsible process in atherothrombosis [2]. Assessment of platelet function with MPV level gained popularity in recent years. MPV level reflects the platelet production rate and activation. It is a more reliable measurement of platelet function than the platelet count alone. Elevated levels of MPV have been demonstrated to be an independent predictor for ischemic vascular events, recurrent MI or death from coronary artery disease [4,20,21]. Higher MPV levels have been also found to be associated with adverse outcome after acute coronary syndrome [22]. Therefore, increased MPV is a potentially useful biomarker for thromboembolic complications in cardiovascular disorders.

Numerous factors such as hypothermia, shear forces, exposure to artificial surfaces, the use of exogenous drugs and the release of endogenous chemicals may cause platelet activation during CPB [23]. Proteins, such as P-selectin are expressed by activated platelets. P-selectin is a molecule that contributes to the development of thrombus [24]. Thrombus formation is aggravated by this mechanism in the course of CPB despite the decrease in platelet count. Furthermore, activated platelets attach to the vascular endothelium and causing the expression of adhesion molecules [25]. This produces a cascade of immunologic reactions, and these processes in molecular level may be a key point of clinical adverse events with the contribution of platelet activation.

The potential role of platelets has been evaluated for the bleeding complication after cardiac surgery [26]. However, there are limited data emphasizing the association of platelet activation with adverse outcomes following CABG [27]. Steele et al. have established a relationship between decreased platelet survival and saphenous vein graft occlusion [28]. Similarly, Tavil et al. have demonstrated that there was a significant increase in MPV levels in patients with saphenous vein disease [7]. All of these findings are associated with late-phase graft occlusion and suggests that activation of platelets may have a contribution to this outcome.

Preoperative MPV level over 8.75 fL was associated and a strong independent predictor only with combined adverse events, postoperative MI and MACE. These outcomes of the study population were mainly composed of events that may be associated with thrombosis such as postoperative MI, reoperation, early repeat revascularization and mortality. This finding may indicate that the increased MPV value may show predisposition to thrombosis.

There are a vast number of studies that have shown the predictor value of anemia in cardiac surgery patients [9,16,17]. Low levels of hematocrit could result in worsening of symptoms in cardiovascular patients. Decrease in hemoglobin level may lead to limited compensation through increased heart rate and stroke volume [29]. However, although the increase in post-CABG adverse events was independently associated with low preoperative hematocrit levels, cardiac adverse events were caused by other factors frequently present in anemic patients. Therefore this study was designed with patients who had hematocrit level above 30% or hemoglobin level over 10.0 g/dl. In our study, it was shown that there is a relationship between the preoperative hematocrit levels and post-CABG adverse events even in non-severe anemic patients.

Preoperative hematocrit level less than 41.5% was associated with combined adverse events, postoperative MI, MACE, re-hospitalization and prolonged stay of ICU and hospital. However it was a strong independent predictor only for combined adverse events and prolonged stay of ICU and hospital. The higher unadjusted morbidities such as mentioned above among patients with low hematocrit levels is probably the result of older age, female predominance and higher comorbidities.

All of the other hematologic parameters (including differential leukocyte count and N/L ratio) showed significant relation neither with post-CABG adverse events nor with mortality. Conversely, Gibson et al. demonstrated that the preoperative N/L ratio is a predictor of adverse outcome after CABG [19]. Also there are reports regarding neutrophil and lymphocyte components as a predictor of outcome in ischemic heart disease [30,31].

The multiple logistic regression models clearly demonstrate that the patients who come up against adverse events after CABG have significantly higher preoperative MPV levels and lower preoperative hematocrit levels compared to the patients with uneventful course. Furthermore, this finding indicates the independent predictive value of the preoperative MPV and hematocrit levels with post-CABG adverse events.

The use of combined adverse events provides an objective measure of outcome. However, the present study has some limitations. Firstly, this is a single center experience representing a relatively small numbers of patients. All analyses were limited to short-term and operative outcomes. Analysis with long-term follow-up data would have more reliable findings. The MPV levels could be affected by platelets becoming smaller than expected when exposed to acetylsalicylic acid. Acetylsalicylic acid usage in coronary artery disease patients prior to operation might interfere with the platelet shape and might lead to different results. There could be an association between transfusion amounts that would be more prominent in lower hematocrit levels with the adverse events. The impact of transfusion during surgery or postoperative period was not included in the analyses. The different variables between the groups (male gender, EF, NYHA class, EuroSCORE, time on bypass and number of bypass grafts) were evaluated in multivariable models together with the MPV and hematocrit levels to avoid the inaccurate impact on the results. The postoperative values of the MPV and hematocrit were not included in this study. Since the cause-and-effect relationship of these measures and the outcome might be variable and we thought that this might be a subject of a different study.

## Conclusions

To the best of our knowledge, this is the first study that the MPV level is evaluated for the adverse outcomes after CABG. The current data show a clear association of preoperative MPV and hematocrit levels with post-CABG adverse events. The prognostic value of these measures is independent of other well-defined individual risk factors. In contrast, neither the N/L ratio nor the WBC count including differential leukocyte count demonstrated a significant relation with post-CABG adverse events. The MPV level along with hematocrit levels, which are readily available, may play a role in risk stratification for patients undergoing CABG. However, it is not clear if these measures have a direct contribution to outcomes, so further investigations are required to explain the underlying mechanisms.

## Abbreviations

CABG: Coronary artery bypass grafting; EuroSCORE: European System for Cardiac Operative Risk Evaluation; MACE: Major adverse cardiac events; MI: Myocardial infarction; MPV: Mean platelet volume; N/L: Neutrophil to lymphocyte (N/L)

## Competing interests

The authors declare that they have no competing interests.

## Authors’ contributions

EUU carried out the conception and design. EUU, SK, ABD and CLB carried out the analysis and interpretation. EUU, ST, MS and ÜK participated in the data collection. EUU, AO, SK and ABD participated in the writing the article, the critical revision of the article and the statistical analysis. All authors read and approved the final manuscript.
